# Effect of different conditions on the germination of coix seed and its characteristics analysis

**DOI:** 10.1016/j.fochx.2024.101332

**Published:** 2024-03-28

**Authors:** Lidan Dong, Yun Yang, Yongcai Zhao, Zhengyu Liu, Cuiqin Li, Laping He, Lihua Liu

**Affiliations:** a Key Laboratory of Agricultural and Animal Products Store & Processing of Guizhou Province, Guizhou University, Guiyang 550025, PR China; b College of Liquor and Food Engineering, Guizhou University, Guiyang 550025, PR China; c School of Chemistry and Chemical Engineering, Guizhou University, Guiyang 550025, PR China; d Key Lab of Fermentation Engineering and Biopharmacy, Guizhou University, Guiyang 550025, PR China; e Guizhou Nanfang Dairy Co, Ltd, Guiyang 551499, PR China

**Keywords:** Coix seed, Germination, Uniform design, γ-Aminobutyric acid, Flavor change

## Abstract

Coix seed (CS) has high nutritional value, but the deep processing of CS is relatively limited. Sprouting can significantly improve nutritional value, laying the foundation for efficient consumption or further processing. The optimal conditions for the germination of CS are a soaking temperature of 36 °C for 10 h and a germination temperature of 29 °C for 24 h. Under these conditions, the final germination rate of CS reached 90%. Additionally, the content of γ-aminobutyric acid was 21.205 mg/100 g; soluble protein, free amino acids, γ-aminobutyric acid, and other essential substances increased in CS. Especially after germination, the γ-aminobutyric acid (GABA) content increased by 7.8 times compared with the GABA content of ungerminated CS. Therefore, the nutritional value and flavor of germinated CS are better than those of ungerminated ones, which establishs a solid foundation for its application in developing various products such as compound health drinks, coix yogurt, and others.

## Highlights

• The germination rate of coix seed (CS) reached 90% through uniform design.
• Germination improved theγ-aminobutyric acid content in CS by 7.8 times.
• Germination increased soluble protein and free amino acid content in CS.
• The overall nutritional value of CS was enhanced as a result of germination.

## Graphical abstract

**Figure:**
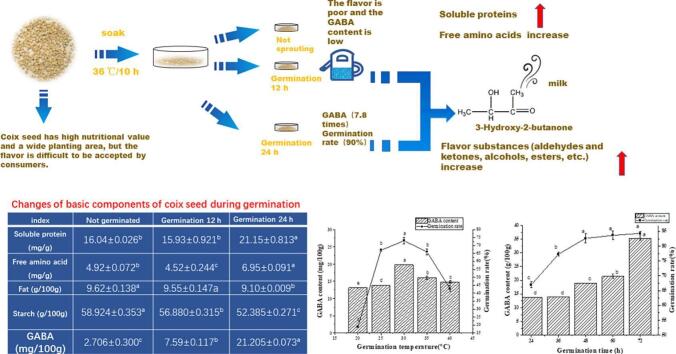

## 1 Introduction

In food development, there is a growing interest among people in green and healthy food options, particularly those rich in biological activity (Mihaela et al., 2023). Coix seed (CS) is a unique agricultural product in Guizhou Province, known for its rich bioactive substances, such as polysaccharides, coix, phenols, steroids, and lactams. As raw materials of functional foods (Wei et al., 2023), it has significantly contributed to the economic development of agricultural products in Guizhou Province (Tan, 2022). So far, numerous studies have demonstrated the positive effects of CS and its extracts on human health, including antioxidant activity, prevention of osteoporosis, anti-tumor, and anti-obesity (Anyan et al., 2021). In earlier studies, CS was primarily used for simple daily cooking or direct consumption, limiting the variety of well-processed products and not fully utilizing its high nutritional value. CS germination converts the macromolecules into easily usable small molecules and produces new high-value components such as γ-aminobutyric acid (GABA). GABA is a non-protein amino acid that is present in both animals and plants. GABA's main production pathway involves the glutamic acid's decarboxylation catalyzed by the enzyme glutamic acid decarboxylase (GAD) (Cho & Lim, 2018). Its primary function in animals is inhibitory neurotransmitters. It can trigger various metabolic processes, including calming and relieving excitement, lowering blood pressure (Thuwapanichayanan, Yoosabai, Jaisut, Soponronnarit, & Prachayawarakorn, 2015) and cholesterol, and improving human health (Wang et al., 2015). Food rich in γ-aminobutyric acid (GABA) is indeed considered a functional food, and its development has emerged as a significant research direction in the field of functional foods in recent years. Therefore, germinated CS is an excellent raw material for producing functional foods such as beverages, and it is expected that the health benefits of sprouted CS will also stimulate the interest of food producers and consumers.

In the humid places of ancient warehouses, rice “sprouting” often occurred. Rice is typically dried or sun-dried to inhibit its growth and prolong its storage life. From the biological point of view, germination refers to a series of orderly physiological and morphological changes in grain from water absorption and expansion (Chen, 2020). Current research indicates that the germinated grain or bud has higher nutritional and physiological value than the non-germinated grain (Donkor, Stojanovska, Ginn, Ashton, & Vasiljevic, 2012). On this basis, germinated CS as a new type of food has received widespread attention. Cunzhi Li successfully transformed CS into a milky white beverage through enzymolysis, resulting in a delightful combination of milk flavor and a distinct sweet taste (Li, Huang, Yan, Fu, & Liu, 2010). Xu Lei's study on the changes in nutrient composition, physicochemical properties, and biological activities of CS during germination showed that red CS had higher antioxidant activity in vitro.

Meanwhile, the red CS was significantly superior to the white **(Lei et al., 2017). Igbokwe Chidimma Juliet reviewed the research on the physical and chemical composition, biological activity, processing, application, and safety of coix seed, which also made us fully understand that there are still many shortcomings in the development of the coix seed industry at this stage (Juliet et al., 2022). In addition, functional molecules with important biological activities, such as lysophosphatidylcholine, coix seed, 10-formyltetrahydrofolic acid, oleanolic acid, gallocatechin, chlorogenic acid, and eugenic acid, were significantly enriched after germination. Although germination benefits the increase of nutrients in CS, the research still has several problems. Firstly, there is a lack of studies on the composition and functional changes of bioactive ingredients during the germination process; secondly, the control of pre-germination conditions and the mechanism underlying the post-germination flavor of CS are areas that require further investigation. One study found that a considerable number of hormone-signaling genes were significantly up-regulated during seed germination, which activated multiple metabolic processes. Meanwhile, the results of the multi-omics joint analysis of this study showed that glucose and fatty acid metabolism played an essential role in the process of seed germination under hormone regulation (Donghao et al., 2023); finally, the germination mechanism of CS lacks in-depth and systematic study to establish a consistent scientific consensus; meanwhile, there is a limited availability of germinated products.

Germination requires appropriate temperature, humidity, oxygen, and other external conditions to support normal physiological metabolism and growth. The benefits of germination are worth exploring. For instance, a study found that the lipid-lowering and antioxidant functions of germinated CS were better than those of non-germinated CS (Lei et al., 2017). There was also evidence that germination improved the flavor of grains (Kornelia, Chandra-Hioe, & Damian, 2018). Therefore, to enhance the functional characteristics and flavor components of food, study the changes in flavor and function of CS during the germination process, use the attributes of germinated CS to design efficient and high-value products, realize the rational use of resources, and finally achieve the goal of industrialization. As a normal growth phenomenon, germination runs through the whole life course of organisms. By changing the germination conditions, the nutritional value of the barley can be significantly improved, and it will not harm human health, laying a foundation for the development of healthy food (Dekka, Vincent, Tito, Jagan, & Rajagopal, 2023). The main objective of this study is to assess the germination rate and γ- amino butyric acid content. Secondly, taking the seed germination rate of CS as the evaluation index, the optimum technological parameters were determined by uniform design. Lastly, the changes in physicochemical indexes and flavor of CS before and after germination were analyzed, laying a foundation for developing germinated CS food.

## 2 Materials and methods

### 2.1 Experimental materials

#### 2.1.1 Materials

CS was provided by Guizhou Renxin Agricultural Development Co., LTD (Guizhou, China), and was cultivated using organic and compound fertilizers (nitrogen, phosphorus, and potassium) while adhering to insecticide-free cultivation practices.

#### 2.1.2 Reagents

GABA standard, 2, 4-dinitrofluorobenzene (FDNB) were from Shanghai Maclin Biochemical Co., LTD., China. Glutamic acid standard, Bovine Serum Protein, and Coomassie Bright Blue G-250 were from Shanghai Solarbio Biotechnology Co., LTD. China. Phenol, glucose, and sodium bicarbonate were from Sinophosphoric Group chemical reagent Co., LTD., China. Standard n-decane was from Shanghai Yi En Chemical Technology Co., LTD., China. Sulfuric acid was from East Sichuan Chemical Industry Co., LTD., China.

### 2.2 Experimental method

#### 2.2.1 Preparation of germinated CS

According to (Meng, 2009), the germination method was modified to suit the experiment's needs. Initially, CS was chosen and immersed in a 1% sodium hypochlorite solution for 15 min, followed by rinsing with deionized water until the smell of sodium hypochlorite was no longer detectable. The sterilized CS was soaked in 10 times the volume of deionized water to ensure full moisture absorption. After soaking, CS was soaked in 1% sodium hypochlorite solution for 15 min, then washed with deionized water until there was no sodium hypochlorite taste. Then lay the CS between two layers of gauze (sterilized at 121 °C for 20 min), place it in a temperature and humidity-controlled incubator (HSP-80B, Tianjin Sedaris Experimental Analytical Instrument Factory, China) to germinate, and spray water every 12 h to keep it moist. After germination, the germinated CS were washed with deionized water and then dried in an oven (GZX-9070, Shanghai Boxun Industrial Co., LTD., China) at 40 °C for half an hour to terminate the activity. Then dry it to make the final moisture content about 10% ∼ 12%, take out the grinding powder, pass the 80 mesh sieve, and store it at −20 °C for testing. Three replicates were established for each group of tests.

#### 2.2.2 Determination of GABA content

The GABA content was determined using HPLC (U3000,Thermo Fisher, USA) with 2, 4-dinitrofluorodinitroben-zene (FDNB) pre-column derivatization (Zhang, 2020).

Chromatographic conditions: ^Acculam^™ 120 C18 column (inner diameter 4.6 × 250 mm, particle size 5 um), detector (UltiMate 3000, Thermo Fisher Technologies, USA) wavelength 370 nm, column temperature 35 °C, analysis time 10 min, flow rate 1 mL/min, injection volume 10 μL. Mobile phase acetonitrile: phosphoric acid aqueous solution (volume fraction 0.12%) =50:50.

#### 2.2.3 Headspace solid-phase microextraction analysis of volatile flavor compounds in germinated CS

The method outlined in the reference was slightly modified (Rami et al., 2021).

Sample treatment and extraction conditions: 1.5 g of the sample was added to a 22 mL headspace bottle, which was quickly sealed with a gasket (PTFE), and then 20 μL of n-decane (ether as solvent) at 0.75 mg/mL was introduced as an internal standard. It was closed for 15 min at 60 °C. The aged extraction head was inserted into the headspace of the sample bottle and adsorbed at 45 °C for 60 min. After adsorption, the extraction head was placed into the gas chromatography (6980, Agilent Technology Co. LTD., USA) sample inlet and analyzed at 250 °C for 5 min. Then, the instrument was started to collect data. At the same time, three parallel groups were set up for each experimental group to repeat the experiment to ensure its accuracy.

Gas phase conditions: Agilent 19091S-436 HP-5MS capillary column was used, and the column temperature box: the initial column temperature was 45 °C, maintained for 1 min, first rose to 220 °C at the rate of 5 °C/min, then rose to 250 °C at the rate of 8 °C/min, the carrier gas was helium (1 mL/min), and the split ratio was 20:1. The injector and detector temperatures were set at 250 °C and 280 °C, respectively.

Mass spectrum conditions: electron bombardment ion source (EI), electron energy 70 eV. The ion source temperature was set at 230 °C, and the interface temperature was maintained at 250 °C.

The identification method for volatile compounds involved comparing their mass spectra with the standard spectra in the MS library of the National Institute of Standards and Technology (NIST2020. L). The content of each volatile substance in the sample was calculated according to the internal standard method of (Zhou, Chong, Ding, Gu, & Liu, 2016). Qualitative analysis was conducted by comparing each component's retention index and mass spectrum.

#### 2.2.4 Determination of physical and chemical indexes

Soluble protein: Coomassie brilliant blue G-250 staining method (Yang, 2018);

Fat content: Soxhlet extraction method (Fat analyzer, SER148, Jiasheng (Hong Kong) Technology Co., LTD., China) referred to Pattra's method with slight improvement (Pattra, Suvarnakuta, Chutinun, Sunthorn, & Kaemwich, 2016).

Starch content: anthrone colorimetry (Ultraviolet Spectrophotometer, T6 New Century, Beijing Puyang General Instrument Co., LTD., China) (Guo & Peng, 2007);

Free amino acid: ninhydrin colorimetry, referred to J. Xu's method (Xu, Weilong, Xiaolei, Jing, & Haitao, 2013);

Germination rate: three parallel lines were set, each with 100 grains. After germination under specific conditions, the germinated grains were calculated. The formula for calculating the germination rate of CS was as follows:

$$\text{Germination rate}\left(\%\right)=\frac{\text{Number of sprouted}\;\mathrm{CS}-\mathrm{CS}}{\text{Total number of grains of}\;\mathrm{CS}-\mathrm{CS}}\times 100\%$$

#### 2.2.5 One-factor-at-a-time (OFAT) experiments

The initial germination conditions of the CS were as follows: soaking time of 12 h, soaking temperature at 25 °C, germination time of 24 h, and germination temperature at 25 °C, soaking temperature (20 °C，25 °C，30 °C，35 °C，and 40 °C), soaking time (4 h，8 h，12 h，16 h，and 20 h), germination temperature (20 °C，25 °C，30 °C，35 °C，and 40 °C) and germination time (24 h，36 h，48 h，60 h，and 72 h) were selected successively for optimization.

#### 2.2.6 Determination of optimum germination conditions

Based on the One-factor-at-a-time (OFAT) experimental results, the soaking temperature (X_1_), soaking time (X_2_), germination temperature (X_3_), and germination time (X_4_) were selected as the influencing factors, with the germination rate of CS serving as the index. The influence of different factors on the index was analyzed using a uniform design of 4 factors and 5 levels. The level of uniform design factors is shown in **Table S1**. Then, a U * 15 (15^7^) uniform design table was selected to arrange the experiment, as depicted in **Table S2.**

The seed germination of CS was conducted based on the experimental plan outlined in the uniform design table. With the germination rate after germination as the index, SPSS 25.0 was used to perform the progressive regression analysis method on the obtained experimental data to establish the mathematical model of seed germination rate. The soaking time, soaking temperature, germination time, and germination temperature during germination were included, and the programming solver in Excel software was used to formulate the optimal combination of the regression equation for seed germination rate. The predicted germination rate of CS was then calculated.

#### 2.2.7 Verification test

The experiment was performed according to the optimal combination obtained from the regression equation, and the experimental results were compared with the theoretical value to verify whether the germination rate of CS could reach the predicted value under the optimal germination conditions. This was done to judge whether the optimal culture combination was accurate and reliable.

#### 2.2.8 Data analysis

All of the tests were conducted in triplicate. The data are displayed as the mean ± standard deviation (SD). One-way ANOVA analysis was performed utilizing SPSS 22 (SPSS Inc., Chicago, IL, USA). Differences among groups were assessed using Tukey's HSD test, with *p*-values <0.05 deemed statistically significant.

## 3 Results and discussion

### 3.1 Effect of germination conditions on CS

#### 3.1.1 Effect of soaking temperature on CS

**Fig. 1:**
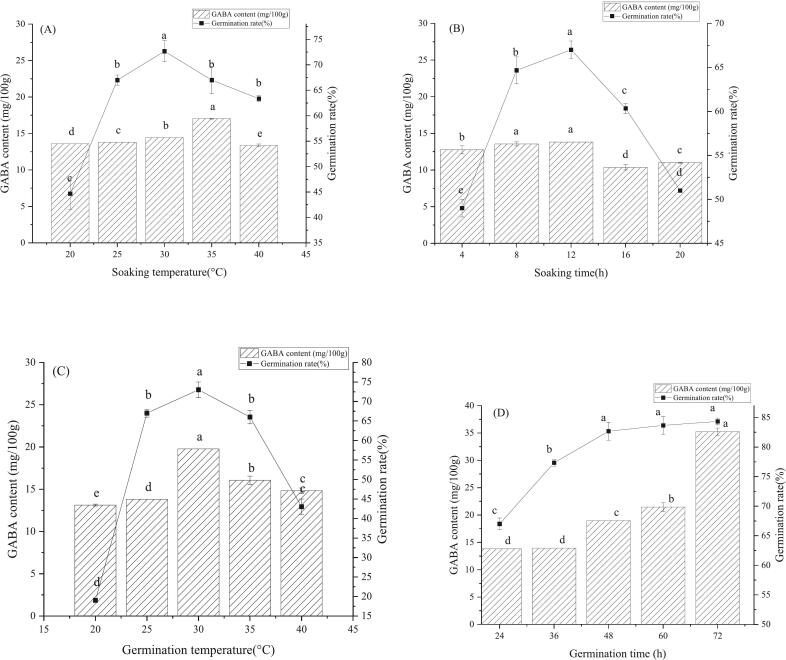
Effect of germination conditions on germination rate and GABA content of CS: (A) soaking temperatures, (B) soaking times, (C) germination temperatures, and (D) germination times. The average value with different lowercase letters showed a significant difference (*P* < 0.05).

The impact of soaking temperature on the germination of CS is crucial and requires further investigation, as different types of grains have specific temperature requirements for germination and growth. As a small molecular weight non-protein amino acid, GABA has physiological effects during germination, such as improving sleep quality and lowering blood pressure. The soaking temperature significantly impacts the content of GABA in sprouted grain. The experimental results of some researchers prove that appropriate soaking temperature will promote the accumulation of GABA in germinated grains (Chen et al., 2018; Ferreira et al., 2018). In Fig. 1A, on the one hand, as the soaking temperature gradually increased, the germination rate showed a trend of rapid rise at first and then a slow decline. When the soaking temperature was <25 °C, the germination rate was low, indicating that low temperature had a greater effect on germination and inhibited the growth of grain germ. The germination rate of CS reached its peak when the soaking temperature was 30 °C. However, the germination rate of CS dropped significantly when the temperature ranged between 35 °C and 40 °C. The high temperature will accelerate the metabolic rate of CS and cause excessive consumption of dissolved oxygen in the soaking water. As a result, seed embryos of CS will be damaged, and the growth of radicle and hypocotyl will be deactivated, thus inhibiting seed germination. Eventually, the germination rate decreases.

Secondly, when the soaking temperature continued to increase, the content of GABA initially rose to the maximum and then decreased slowly. After being soaked in water at temperatures ranging from 20 to 30 °C for 12 h, there was a slight increase in the content of GABA in germinated CS; however, the change was not noticeable. However, the content of CS proliferated when the soaking temperature was 30–35 °C and reached the maximum of 17.02 mg/100 g at 35 °C. Following soaking in water at 40 °C, the content of GABA in germinated CS decreased significantly. The reason is that some macromolecular substances in CS are decomposed into small molecular substances under the action of endogenous enzymes, thus providing a particular substrate for the production of GABA, which provides a material basis for maintaining certain life activities (Xu, 2019). Besides, with the continuous rise of soaking temperature, even higher than the optimum temperature of the endogenous enzyme, the activity of this enzyme is inhibited; for example, the inhibition of endogenous glutamic acid decarboxylase (GAD) activity will result in the inability to produce GABA, which will reduce its content. The increase in soaking temperature resulted in an acceleration of the water absorption rate of CS. When its water absorption is too much to reach saturation or even expansion, the cell structure of the CS will break, making the outflow of water-soluble substances such as glutamic acid cause losses. At the same time, when the soaking temperature is higher than the optimum temperature of the endogenous enzyme, its activity will be inhibited. Inhibiting endogenous glutamic acid decarboxylase (GAD) activity can prevent GABA production, reducing its content in the germinated CS. Finally, some research results show that increased GAD activity and germination rate can increase GABA content (Hoang, Thi, Phan, & Van, 2021). The experimental results suggested that soaking temperatures in the range of 25–35 °C aligned with the theoretical requirements for optimal outcomes in the germination process of CS.

#### 3.1.2 Effect of soaking time on CS

The primary conditions for seed germination include sufficient water, proper temperature, and light (Ali et al., 2021). Fig. 1B shows the changes in seed germination rate and GABA content of CS under different soaking times. First, with the extension in soaking time, the germination rate rapidly rose and then rapidly declined. When the soaking time was 12 h, the germination rate of the CS reached the maximum value. The reason is that CS needs appropriate time to absorb sufficient water for germination, and activating some internal enzymes can increase the germination rate (Yang et al., 2021). In contrast, the germination rate decreased rapidly when the soaking time was over 12 h. The reason for this is that when the water in the CS reaches saturation, excessive absorption will destroy the internal structure of the cells of the CS, resulting in a loose tissue structure. Some water-soluble substances necessary for germination are extracted from the cells, which hinders the CS's germination and decreases the germination rate.

Next, with the increase of soaking time, the content of GABA in germinated CS increased first, then decreased, and subsequently increased. In the 4–12 h, the content of GABA increased markedly with the increase of soaking time. When the soaking time was 12 h, the GABA content in the CS peaked at 13.8 mg/100 g. The content of GABA dropped sharply after the soaking time of 12–16 h. However, after the soaking time of 16–20 h, the content of GABA exhibited a gradual increase but remained lower than the maximum value observed. The research results of this experiment were consistent with those of the predecessors (Huang et al., 2021). The reason for this is that during the soaking process of CS, the relevant enzymes required for the synthesis of GABA are activated so that the content of GABA can be increased for some time.

Meanwhile, in the soaking process, the substances in the dried CS changed from gel state to sol state. The dry meanings in the dried CS also changed into soluble substances, which accelerated the metabolic rate of the CS, promoted the generation of endogenous glutamate decarboxylase, and finally increased the content of GABA. However, when the soaking time was 12–16 h, excessive water absorption by the cells caused them to expand and rupture, resulting in significant damage to the cell structure. A large amount of soluble protein flows out, and the enzyme activity is reduced, thus reducing the substrate and enzyme that can provide GABA production and reducing the content of GABA.

#### 3.1.3 Effect of germination temperature on CS

Temperature, as one of the essential factors affecting the germination of CS, is also considered in this experiment. For example, the best temperature value during germination and the acceptable germination rate temperature range (Guangyao, Amber, Von Mark, Claire, & David, 2020). Fig. 1C shows the changes in seed germination rate and GABA content of CS under different germination temperatures. First, the germination rate increased and then decreased with the increase of germination temperature. The low germination rate was observed at 20–25 °C, indicating that low temperature significantly impacted CS germination. The germination rate peaked at 30 °C and decreased as the temperature rose above 30 °C.

Next, as the germination temperature increased, the content of GABA in germinated CS initially rose to a maximum level and then decreased. When the germination temperature was 30 °C, the GABA content in the CS reached its highest level at 19.77 mg/100 g. When the germination temperature was 35 °C or 40 °C, the GABA content was higher than 20 °C or 25 °C. The low temperature was not conducive to the increase of GABA content during the germination of CS, which inhibited the activity of related enzymes and hindered the germination process. The research shows that the reaction of a glutamic acid decarboxylase to produce GABA is irreversible (Agung, Suppasil, & Dietmar, 2020), and the formation of GABA is an enzymatic reaction process in disguised form, which is greatly affected by temperature. When the temperature reaches the optimum level, its content matches the peak; when the temperature becomes excessively high, the protein begins to denature, resulting in a slowdown of the reaction process, ultimately leading to a rapid decline in its content.

#### 3.1.4 Effect of germination time on CS

Fig. 1D depicts the impact of varying germination durations on the germination rate and GABA content of CS. First, as the germination time extended, the germination rate of CS exhibited a gradual increasing trend. When the germination time was 24–36 h, the germination rate increased rapidly; when the germination time exceeded 36 h, the germination rate rose slowly. If the germination time is too long, the dried barley germ will fall off easily, affecting the subsequent experiment, so it is necessary to arrange the germination time reasonably. Secondly, it was evident from the figure that the GABA content had been increasing. Some research findings suggest that prolonging the germination time is beneficial for the production of GABA (Kamjijam, Bednarz, Suwannaporn, Jom, & Niehaus, 2020). Since the germination time was 24 h or 36 h, the content of GABA in germinated CS did not change significantly. When the germination time was 72 h, the content of GABA in germinated CS reached the highest value of 35.23 mg/100 g. The results showed that during the germination process, the original protein in CS could not only be hydrolyzed to provide a sufficient substrate for subsequent glutamic acid decarboxylase, promoting the increase of its GABA content, but also be conducive to the activation of some enzymes in the reaction process, ultimately producing more target products.

### 3.2 Analysis of the results of optimizing the germination conditions of CS by uniform design

#### 3.2.1 Experimental results of uniform design

The results of optimizing the germination conditions of CS by uniform design method are shown in Table 1. The data processing software SPSS was used to conduct multiple stepwise regression analyses of the experimental results. The results of the variance analysis of the regression model are shown in Table 2. The determination coefficient R^2^ = 0.948, the adjusted correlation coefficient R^2^ = 0.933, F = 62.280, *P* = 0.000, indicating that the experimental results were statistically significant, showing that the equation was well fitted with the experimental data, that is, the equation could well fit the germination process of CS and the regression equation had a significant linear relationship.

**Table 1:** Uniform design results.

number	Soaking temperature X_1_ (°C)	Soaking time X_2_ (h)	Germination temperature X_3_ (°C)	Germination time X_4_ (h)	Germination percentage (%)
1	1(24)	5(16)	9(33)	13(48)	85 ± 2.00d^e^
2	2(27)	10(16)	2(27)	10(72)	95 ± 1.00^a^
3	3(30)	15(16)	11(24)	7(36)	87 ± 2.00^d^
4	4(33)	4(14)	4(33)	4(60)	87 ± 0.58^d^
5	5(36)	9(14)	13(30)	1(24)	86 ± 1.53^d^
6	6(24)	14(14)	6(24)	14(60)	93 ± 1.00^ab^
7	7(27)	3(12)	15(36)	11(24)	73 ± 1.00^g^
8	8(30)	8(12)	8(30)	8(48)	95 ± 0.58^a^
9	9(33)	13(12)	1(24)	5(72)	91 ± 1.00^bc^
10	10(36)	2(10)	10(36)	2(36)	79 ± 1.00^f^
11	11(24)	7(10)	3(30)	15(72)	91 ± 1.00^bc^
12	12(27)	12(10)	12(27)	12(36)	90 ± 1.53^c^
13	13(30)	1(8)	5(36)	9(60)	85 ± 0.00^de^
14	14(33)	6(8)	14(33)	6(24)	84 ± 1.73^e^
15	15(36)	11(8)	7(27)	3(48)	94 ± 1.53^a^

Note: The average value with different lowercase letters shows a significant difference (*P* < 0.05).

**Table 2:** Variance analysis of germination rate.

Model	Sum of squares	df	mean square	F	P	R^2^
regression	1543.387	10	154.339	62.28	0.000b	0.948
residual	84.257	34	2.478			
total	1627.644	44				

From Table 3, the regression equation of the germination rate (Y) of CS to the independent variables of soaking temperature (X_1_), soaking time (X_2_), germination temperature (X_3_), and germination time (X_4_) is as follows:

$$Y=-285.068+19.077X_{1}+17.331X_{2}-0.208X_{1}^{2}-0.417X_{2}^{2}-0.071X_{3}^{2}+0.002X_{4}^{2}-0.363X_{1}X_{2}-0.045X_{1}X_{4}+0.109X_{2}X_{3}+0.042X_{3}X_{4}$$

**Table 3:** Parameter table.

	No standardized coefficient		Standard coefficient	T	Significance
	B	Standard error	Beta		
constant	−285.068	49.276		−5.785	0
X_1_	19.077	2.294	13.458	8.314	0
X_2_	17.331	3.101	8.151	5.588	0
X_1_^2^	−0.208	0.027	−8.83	−7.595	0
X_2_^2^	−0.417	0.071	−4.733	−5.854	0
X_3_^2^	−0.071	0.005	−3.002	−13.749	0
X_4_^2^	0.002	0.001	0.617	2.733	0.001
X_1_X_2_	−0.363	0.047	−5.065	−7.706	0
X_1_X_4_	−0.045	0.005	−3.611	−9.028	0
X_2_X_3_	0.109	0.021	1.517	5.191	0
X_3_X_4_	0.042	0.004	3.428	11.03	0

B Prediction variables: (constant), X_3_X_4_, X_2_, X_3_X_3_, X_1_X_1_, X_4_X_4_, X_1_X_4_, X_2_X_3_, X_1_X_2_, X_2_X_2_, X_1_.

First, suppose the coefficient in front of the factor in the linear regression equation is positive. In that case, it has a positive growth effect on the dependent variable Y. Conversely, if the coefficient is negative, it has a negative growth effect on Y. Finally, it can be concluded that the germination time positively impacts the germination rate of CS, indicating that if the germination time is extended, the germination rate of CS will also increase. This conclusion is supported by the report (Singh, Sharma, & Singh, 2018). The regression equation was solved through Excel, and the optimal solution for each factor was finally obtained: X_1_ = 36 °C, X_2_ = 10 h, X_3_ = 29 °C, X_4_ = 24 h. When the obtained value was brought into the equation, Y = 96% was obtained. Therefore, the optimal combination obtained by uniform design was: when the soaking temperature was 36 °C, the soaking time was 10 h, the germination temperature was 29 °C, and the germination time was 24 h, the predicted germination rate was 96%. The optimal combination obtained through uniform design reveals that the value of each factor in the optimal combination differs from the optimal result of OFAT experimentation. The OFAT experiment has mutual promotion and inhibition, so it should be analyzed in the actual situation.

#### 3.2.2 Validation experiment

Under the aforementioned technological conditions, repeated experiments were carried out three times to verify the experimental results, and the conclusion was that the germination rate of CS was 90%, which was close to the predicted parameter value, proving that the model was feasible.

### 3.3 Effect of germination on the content of various components of CS

Under the conditions of uniform design and optimum germination process, the chemical components of CS in different germination stages were determined. The contents of soluble protein, free amino acid, fat, starch, and GABA in CS were significantly affected during germination. First, as shown in Table 4, the soluble protein and free amino acid content of CS decreased slightly within 12 h and increased significantly within 12–24 h. The loss of dry weight in protein, particularly the depletion of carbohydrates through respiration during germination. The free amino acid content increases due to enzyme activation, hydrolyzed protein production, and non-protein nitrogen (Wipada, Han-Seok, & Ya-Jane, 2020).

**Table 4:** Changes of basic components of CS during germination.

index	Not germinated	Germination 12 h	Germination 24 h
Soluble protein (mg/g)	16.04 ± 0.026^b^	15.93 ± 0.921^b^	21.15 ± 0.813^a^
Free amino acid (mg/g)	4.92 ± 0.072^b^	4.52 ± 0.244^c^	6.95 ± 0.091^a^
Fat (g/100 g)	9.62 ± 0.138^a^	9.55 ± 0.147a	9.10 ± 0.009^b^
Starch (g/100 g)	58.924 ± 0.353^a^	56.880 ± 0.315^b^	52.385 ± 0.271^c^
GABA (mg/100 g)	2.706 ± 0.300^c^	7.59 ± 0.117^b^	21.205 ± 0.073^a^

Note: The average value with different lowercase letters shows a significant difference (*P* < 0.05).

Concurrently, the fat content of CS was between 9.62% and 9.10%. Unripe CS fat content was higher but decreased significantly after 24 h of germination. Moreover, the content of starch gradually reduced from 58.92% to 52.38%, which was because the fat and starch in CS were degraded to provide the energy needed for the growth of its embryo during germination, which reduced its content (Xu et al., 2017). In conclusion, the GABA content of germinated CS was significantly increased, 7.8 times higher than that of non-germinated CS. The reason is that the hydrolase decomposes the polymer, resulting in the production of a large number of bioactive substances.

### 3.4 Effect of germination on the volatile flavor of CS

Headspace solid-phase microextraction and gas chromatography–mass spectrometry (GC–MS) were used to analyze the volatile flavor compounds during various stages of CS germination under optimum conditions. At first, the types of volatile flavor components obtained through GC–MS at three different stages of CS germination (no germination, 12 h in germination, and after germination) are summarized as shown in **Table S3**.

The volatile components of germinated CS samples at different stages differed significantly. As shown in **Table S4**, first of all, 171 volatile flavors were identified in the samples of three stages, including 79 hydrocarbons, 18 aromatics, 13 esters, 16 alcohols, 2 acids, 10 aldehydes, 9 ketones, 4 amines, 2 phenols, 2 ethers, and 16 other compounds. The (+) -limonene in hydrocarbons reached an OAV value of 12.04 at 12 h of germination, which has a pleasant lemon flavor and effectively improves the flavor after germination. Secondly, 88, 117, and 86 volatile flavor compounds were identified from unripe, germinated 12 h, and germinated 24 h. There were 28 kinds of volatile components only in germinated CS; there were 48 kinds of volatile components in CS, which only existed in 12 h of germination; however, 21 volatile components were detected in CS seeds after 24 h of germination but not in the other two stages. During the germination of CS, phenols, ethers, acids, esters, aromatics, and hydrocarbons increased obviously at 12 h and decreased slightly at 24 h. In the aromatics, the OAV value of Cymene with fresh fragrance increased first and then decreased during germination, reaching a peak value of 25.73 at 12 h after germination.

Nevertheless, the overall content increased compared to that before germination. Aldehydes, alcohols, and other substances exhibited an increase 12 h after germination but decreased 24 h after germination, reaching levels lower than those observed before germination. As an aldehyde, N-octanal is a colorless liquid with a strong fruit flavor. The OAV value was too high when not germinated, and decreased from 2159 to 1437 after germination, making the flavor more coordinated. Ketones and amines gradually decreased during germination, but amines were not detected after germination. Finally, most flavors were more abundant at 12 h of germination. The increase in flavor was mainly related to a series of degradation and biotransformation reactions during the germination of the CS.

Lipid oxidation and decomposition mainly produce aldehydes, ketones, and alcohols. Generally, aldehydes usually have a slight green or grass flavor (Gonzalez-Cebrino, Garcia-Parra, Ramirez, & Rosario, 2016). A fat or waxy aroma characterizes unsaturated aldehydes, while ketones impart flavors reminiscent of fruit, herbs, and bananas. Alcohols provide pleasant sweetness and floral fragrance. For example, the OAV value of 1-octene-3-ol decreased significantly 24 h after germination, which contributed to the formation of the final good flavor. These compounds will produce ideal flavor at relatively low concentrations and contribute to rice's fresh and greenish flavor, but at higher concentrations, aldehydes may introduce a peculiar smell (Biao et al., 2019). N-hexanal, no aldehyde, decanal, 2-heptanone, 2-pentylfuran, 1-octen-3-ol, etc., are typically green and beany compounds (Ann-Kathrin, Raphaela, Alessa, Anne, & Yanyan, 2022; Jiang et al., 2016). This typical green and beany smell is difficult to accept by Western consumers, which limits its application and development. It could be observed from Table 5 that most of the ketones decreased significantly during the germination of CS, and the contents of aldehydes and alcohols increased rapidly after 12 h of germination but decreased significantly after 24 h of germination. This conclusion was verified in the study of (Xia, Mei, Yu, & Li, 2017). The sharp decrease in its content is because some compounds or the fatty acids that produce them are metabolized into other compounds or diffused into the water phase during the soaking process. The ethanol content in alcohols increased significantly at 12 h of germination, followed by a decrease at 24 h. Furthermore, ethanol can break dormancy by facilitating the tricarboxylic acid cycle or glycolysis or by changing the properties of the membrane, but its high concentration will also lead to seed germination failure. Therefore, the ethanol content increased in the middle stage of germination and decreased in the later stage, which promoted the germination of CS to a certain extent.

**Table 5:** Volatile Flavors of Germinated CS.

Substance CAS		OAV			Threshold (mg/kg)
		Germination 0 h	Germination 12 h	Germination 24 h	
2-Amino-5-methylbenzoic acid	2941-78-8	0.000245	–	–	151

*aldehyde*					
N-hexanal	66–25-1	0.0127	0.0078	0.0033	25
Benzaldehyde	100–52-7	2.018	2.482	1.585	0.3
n-octanal	124–13-0	2159	2154	1437	0.0001
Phenylacetaldehyde	122–78-1	–	6.668	–	0.009
2-Phenylpropionaldehyde	93–53-8	0.0996	0.2381	–	0.3(beer)
Nonanal	124–19-6	0.00358	0.00597	0.00287	122.45(liquor)
Trans-2-nonenal	18,829–56-6	–	1563	–	0.000065
Decanal	112–31-2	10.056	17.08	8.02	0.005

*Ketones*					
3-hydroxy-2-butanone	513–86-0	–	0.0038	0.0036	10
2-heptanone	110–43-0	0.2677	–	–	0.2

*alcohols*					
ethanol	64–17-5	0.00002	0.00011	0.00005	2900
N-amyl alcohol	71–41-0	0.0189	0.0103	0.0052	5
1-octene-3-ol	3391-86-4	76.09	72.06	35.42	0.002
Isooctanol	104–76-7	0.0064	0.0239	0.0090	16.60
Benzyl alcohol	100–51-6	0.0375	0.0944	–	5.5
N-octanol	111–87-5	1.4965	3.1283	1.4665	0.054
Linalool	78–70-6	–	–	34.647	0.0015

*esters*					
δ- Dodecalactone	713–95-1	–	3.36	–	0.01
Butyl butyrate	109–21-7	–	0.00002	–	10,000

*Aromatics*					
methylbenzene	108–88-3	0.4	0.6325	0.6319	0.14
ethylbenzene	100–41-4	0.9459	1.5295	1.0867	0.78
O-xylene	95–47-6	0.6339	0.8395	0.7811	3.0(Homogenized milk)
M-xylene	108–38-3	0.2382	0.2927	0.2552	5.5(Cookies)
Cumene	98–82-8	0.0881	0.1409	0.1152	0.4(Homogenized milk)
Cymene	99–87-6	15.52	25.73	16.93	0.0133

*Hydrocarbons*					
ethylene oxide	75–21-8	–	0.0003	–	905
N-hexane	110–54-3	–	0.0969	0.1584	0.5(beer)
N-hexadecane	544–76-3	–	0.000348	–	300
chloroform	67–66-3	–	0.0214	0.0216	2.0
Undecane	1120-21-4	0.0083	0.0139	0.0114	16.82
n-Undecane	1120-21-4	–	–	0.0019	16.82
T-tridecane	629–50-5	0.0147	0.0267	0.0140	22.69
Pinene	7785-70-8	2.492	4.151	2.399	0.033
β- Water celery	555–10-2	2.188	–	–	0.036
4(5)-Propene	29,050–33-7	–	–	0.0241	10.93
γ- Terpene	99–85-4	0.0499	0.0857	0.0516	2.14
1-Tridecene	2437-56-1	0.0015	0.0031	–	15.44
(+) - limonene	5989-27-5	6.07	12.04	9.71	0.034

*others*					
2-pentylfuran	3777-69-3	56.05	–	–	0.0048
2,4,5-trimethylthiazole	13,623–11-5	–	1.42	0.57	0.05

Esters help to form pleasant fruit and flower fragrance. Due to their low threshold levels, esters make a noticeable contribution to flavor profiles. Acids, phenols, ethers, and amines were detected in 2, 2, 2, and 4 species, respectively, during the germination of CS. Amines were generally decreased but were not seen 24 h after germination, which was beneficial to the flavor of CS. The acid content was higher than before germination, possibly due to increased free fatty acid levels or the significant growth of lipoxygenase activity stimulated by germination. The content of 2, 6-di-tert-butyl-p-cresol in phenols gradually increased with the germination process. 2, 6-di-tert-butyl-p-cresol is a natural polyphenol that can prevent oxidation and its harmful effects on human health, thus improving the quality and nutritional value of food (Bakhtawar et al., 2020). Eighteen types of aromatic compounds were identified during the germination of CS. Fifteen, thirteen, and thirteen species were identified as unripe, germinated at 12 h, and germinated at 24 h, respectively. Yet, the aromatic substances detected were the same type, mainly from raw materials, so they contributed little to flavor. The findings indicated that germination could significantly affect the flavor components of CS. For example, n-hexanal, nonaldehyde, 2-heptanone, 2-pentylfuran, and other components that will produce an unpleasant taste at higher concentrations will be reduced. Lastly, the added flavor after germination is usually more pleasant.

## 4 Conclusion

This study aimed to investigate the effects of various conditions on the germination of CS. The results showed that the best germination conditions were soaking temperature and soaking time of 36 °C and 10 h, respectively. Under a germination temperature of 29 °C and 24 h, the germination rate reached 90%, and GABA content was 21.205 mg/100 g. Besides, the content of soluble protein, free amino acid, GABA, and other vital substances in CS increased after 24 h of germination, and the GABA content of CS after germination was 7.8 times higher than that of the unripe CS. 171 kinds of volatile flavor substances were identified in the samples of three stages. Overall, the flavor of CS has been significantly enhanced after germination. In summary, this study explored the influence of varying germination conditions on the coix seed, not merely furnishing raw materials enriched with high nutritional value and an enhanced profile of active substances for the subsequent creation of beverages, bread, wine, and other food products, but also establishing a robust foundation for the efficient utilization of cereals.

## CRediT authorship contribution statement

**Lidan Dong:** Data curation, Formal analysis, Methodology, Software, Writing – original draft, Writing – review & editing. **Yun Yang:** Data curation, Methodology, Writing – original draft. **Yongcai Zhao:** Methodology. **Zhengyu Liu:** Methodology. **Cuiqin Li:** Conceptualization, Methodology, Supervision. **Laping He:** Conceptualization, Methodology, Supervision. **Lihua Liu:** Conceptualization, Supervision.

## Declaration of competing interest

The authors declare no competing financial interests.

## Data Availability

The data that has been used is confidential.

## Appendix A Supplementary data

Supplementary material Image 1
