# Supplementary material for: Effect of different conditions on the germination of coix seed and its characteristics analysis
Source: Food Chem X. 2024 Mar 28;22:101332. doi: 10.1016/j.fochx.2024.101332 (PMC10997825; doi:10.1016/j.fochx.2024.101332)
Supplement: Supplementary file 1 — Supplementary material [file mmc1.docx]

**Supplementary materials**

**Table S1** Level Table of Uniform Design Factors

| factor | level | | | | |
| --- | --- | --- | --- | --- | --- |
|  | 1 | 2 | 3 | 4 | 5 |
| X_1_ Soaking temperature °C | 24 | 27 | 30 | 33 | 36 |
| X_2_ Soaking time h | 8 | 10 | 12 | 14 | 16 |
| X_3_ Germination temperature °C | 24 | 27 | 30 | 33 | 36 |
| X_4_ Germination time h | 24 | 36 | 48 | 60 | 72 |

**Table S2** Uniform Design Experimental Scheme

| Test | Soaking temperature °C X_1_ | Soaking time h X_2_ | Germination temperature °C X_3_ | Germination time h X_4_ |
| --- | --- | --- | --- | --- |
| 1 | 1(24) | 5(16) | 9(33) | 13(48) |
| 2 | 2(27) | 10(16) | 2(27) | 10(72) |
| 3 | 3(30) | 15(16) | 11(24) | 7(36) |
| 4 | 4(33) | 4(14) | 4(33) | 4(60) |
| 5 | 5(36) | 9(14) | 13(30) | 1(24) |
| 6 | 6(24) | 14(14) | 6(24) | 14(60) |
| 7 | 7(27) | 3(12) | 15(36) | 11(24) |
| 8 | 8(30) | 8(12) | 8(30) | 8(48) |
| 9 | 9(33) | 13(12) | 1(24) | 5(72) |
| 10 | 10(36) | 2(10) | 10(36) | 2(36) |
| 11 | 11(24) | 7(10) | 3(30) | 15(72) |
| 12 | 12(27） | 12(10) | 12(27) | 12(36) |
| 13 | 13(30) | 1(8) | 5(36) | 9(60) |
| 14 | 14(33) | 6(8) | 14(33) | 6(24) |
| 15 | 15(36) | 11(8) | 7(27) | 3(48) |

**Table S3** Volatile flavor types and total amount of CS during germination

|  | Absolute content（μg/100g) | | |
| --- | --- | --- | --- |
|  | Not germinated | Germination 12 h | Germination 24 h |
| Phenols | 27.915 | 42.340 | 31.427 |
| Ethers | 7.779 | 11.062 | 7.923 |
| Acids | 3.706 | 10.802 | 8.339 |
| amine | 9.683 | 8.690 | 0.000 |
| aldehyde | 168.791 | 225.486 | 114.551 |
| Ketones | 27.079 | 17.521 | 6.489 |
| alcohols | 86.364 | 181.562 | 55.289 |
| esters | 46.062 | 114.545 | 65.780 |
| Aromatics | 479.398 | 647.509 | 540.143 |
| Hydrocarbons | 558.889 | 935.026 | 707.683 |
| other | 53.619 | 70.300 | 22.861 |

**Table S4** Volatile Flavors of Germinated CS(supplement)

|  | CAS | Absolute content (μg/100g) | | | | |  |
| --- | --- | --- | --- | --- | --- | --- | --- |
| substance |  | Germination for 0 hours | Germination for 12 hours | Germination for 24 hours | | |  |
| Phenols |  |  |  |  | | |  |
| 4-sec-Butylphenol | 99-71-8 | — | 14.039±0.081 | — | | |  |
| 2, 6-Di-tert-butyl-p-cresol | 128-37-0 | 27.915±0.102^c^ | 28.301±0.101^b^ | 31.427±0.152^a^ | | |  |
| Ethers |  |  |  |  | | |  |
| Dibutyl ether | 142-96-1 | 7.779±0.012^b^ | — | 7.923±0.018^a^ | | |  |
| N-butyl ether | 142-96-1 | — | 11.062±0.021 | — | | |  |
| Acids |  |  |  |  | | |  |
| 2-Amino-6-methylbenzoic acid | 4389-50-8 | — | 10.802±0.019^a^ | 8.339±0.011^b^ | | |  |
| 2-Amino-5-methylbenzoic acid | 2941-78-8 | 3.706±0.004 | — | — | | |  |
| Amines |  |  |  |  | | |  |
| Cyanuric diamide | 645-92-1 | — | 8.690±0.008 | — | | |  |
| L-pyroglutamine | 16395-57-6 | 3.187±0.004 | — | — | | |  |
| Diethylene glycol amine (DGA) | 929-06-6 | 3.391±0.003 | — | — | | |  |
| N- (iminothioureidomethyl) -Benzamide | 33506-46-6 | 3.104±0.001 | — | — | | |  |
| Aldehydes |  |  |  |  | | |  |
| Hexanal | 66-25-1 | 31.738±0.223^a^ | 19.387±0.145^b^ | 8.136±0.069^c^ | | |  |
| Benzaldehyde | 100-52-7 | 60.540±0.543^b^ | 74.471±0.612^a^ | 47.557±0.345^c^ | | |  |
| N-octanal | 124-13-0 | 21.592±0.164^a^ | 21.538±0.166^a^ | 14.374±0.017^b^ | | |  |
| Phenylacetaldehyde | 122-78-1 | — | 6.001±0.005 | — | | |  |
| 2-phenylpropanal | 93-53-8 | 2.988±0.002^b^ | 7.142±0.021^a^ | — | | |  |
| Nonanal | 124-19-6 | 43.793±0.289^b^ | 73.052±0.622^a^ | 35.179±0.274^c^ | | |  |
| Trans-2-Nonenal | 18829-56-6 | — | 10.162±0.118 | — | | |  |
| Decanal | 112-31-2 | 5.028±0.003^b^ | 8.540±0.006^a^ | 4.010±0.004^c^ | | |  |
| α-methylcinnamaldehyde | 101-39-3 | — | — | 3.002±0.002 | | |  |
| 2-Butyl-2-octenal | 13019-16-4 | 3.112±0.007^b^ | 5.193±0.004^a^ | 2.294±0.002^c^ | | |  |
| Ketones |  |  |  |  | | |  |
| 3-hydroxy-2-butanone | 513-86-0 | — | 3.816±0.005^a^ | 3.618±0.007^b^ | | |  |
| 2-heptanone | 110-43-0 | 5.354±0.004 | — | — | | |  |
| 4-decanone | 624-16-8 | 6.508±0.006 | — | — | | |  |
| 5-undecanone | 33083-83-9 | — | 6.919±0.008 | — | | |  |
| 18-pentatriacontanone | 504-53-0 | — | 3.045±0.011 | — | | |  |
| 4-octanone | 589-63-9 | 4.315±0.005 | — | — | | |  |
| 2-Methyl-5-propylnonane | 31081-17-1 | 6.515±0.016 | — | — | | |  |
| 1-Methyl-2-tetralone | 4024-14-0 | 4.387±0.008 | — | — | | |  |
| 4 '- [ (Trimethylsilyl) oxy] propiophenone | 33342-89-1 | — | 3.741±0.013^a^ | 2.871±0.009^b^ | | |  |
| Alcohol |  |  |  |  | | |  |
| (S) -1, 2-Propanediol | 4254-15-3 | 5.444±0.017 | — | — | | |  |
| Ethanol | 64-17-5 | 5.011±0.014^c^ | 33.062±0.244^a^ | 14.303±0.018^b^ | | |  |
| 3-Methyl-3-buten-1-ol | 763-32-6 | — | — | 3.189±0.005 | | |  |
| N-pentanol | 71-41-0 | 9.465±0.003^a^ | 5.138±0.006^b^ | 2.605±0.011^c^ | | |  |
| 1-Octen-3-ol | 3391-86-4 | 15.218±0.013^a^ | 14.412±0.017^b^ | 7.084±0.010^c^ | | |  |
| Isooctanol | 104-76-7 | 10.546±0.011^c^ | 39.746±0.255^a^ | 14.992±0.019^b^ | | |  |
| Benzyl alcohol | 100-51-6 | 20.607±0.118^b^ | 51.904±0.416^a^ | — | | |  |
| N-octanol | 111-87-5 | 8.081±0.006^b^ | 16.893±0.013^a^ | 7.919±0.007^c^ | | |  |
| Linalool | 78-70-6 | — | — | 5.197±0.004 | | |  |
| 2-cyclohexenol | 822-67-3 | 5.951±0.009 | — | — | | |  |
| Tetrahydrolavandulol | 2051-33-4 | — | 6.601±0.004 | — | | |  |
| Batyl alcohol | 544-62-7 | — | 3.212±0.002 | — | | |  |
| Tert-nonyl mercaptan | 25360-10-5 | — | 3.412±0.005 | — | | |  |
| 1, 10-Decanediol | 3074-71-3 | — | 3.983±0.012 | — | | |  |
| 2-Hexyl-1-decanol | 2425-77-6 | — | 3.200±0.017 | — | | |  |
| (Z)-2-methyloct-3-en-2-ol | 18521-07-8 | 6.042±0.015 | — | — | | |  |
| Esters |  |  |  |  | | |  |
| Ethyl hydrazinocarboxylate | 4114-31-2 | — | 13.877±0.144^a^ | 11.120±0.163^b^ | | |  |
| Silanediol dimethyl ester | 1066-42-8 | 21.692±0.178^a^ | 21.879±0.169^a^ | 16.306±0.112^b^ | | |  |
| Delta-dodecalactone | 713-95-1 | — | 3.360±0.018 | — | | |  |
| Butyl butyrate | 109-21-7 | — | 17.521±0.115 | — | | |  |
| Linalyl butyrate | 78-36-4 | — | — | 7.287±0.016 | | |  |
| Diethyl benzamidomalonate | 16798-45-1 | — | — | 4.396±0.011 | | |  |
| Isooctyl acetate | 103-09-3 | 15.622±0.123^c^ | 28.589±0.231^a^ | 16.211±0.114^b^ | | |  |
| 3-Chlorophenyl tetrahydrofurfuryl succinate |  | — | 16.125±0.126^a^ | 6.103±0.016^b^ | | |  |
| 2, 2, 4-Trimethyl-1, 3-pentanediol diisobutyrate | 6846-50-0 | — | 7.146±0.025 | — | | |  |
| Ethyl 10-undecenoate | 692-86-4 | 5.013±0.069 | — | — | | |  |
| Ethyl 9-hexadecenoat | 54546-22-4 | — | — | 4.358±0.058 | | |  |
| Isoamyl butyrate | 106-27-4 | — | 6.049±0.062 | — | | |  |
| Methyl 3- (phenylcarbamoyl) propionate | 5430-83-1 | 3.735±0.044 | — | — | | |  |
| Aromatic |  |  |  |  | | |  |
| Toluene | 108-88-3 | 5.600±0.146^b^ | 8.855±0.018^a^ | 8.846±0.037^a^ | | |  |
| Ethylbenzene | 100-41-4 | 73.779±0.675^c^ | 119.303±2.341^a^ | 84.766±1.156^b^ | | |  |
| O-xylene | 95-47-6 | 190.191±4.238^c^ | 251.855±5.127^a^ | 234.344±3.189^b^ | | |  |
| M-xylene | 108-38-3 | 131.022±2.168^c^ | 160.963±3.256^a^ | 140.381±4.442^b^ | | |  |
| Cumene | 98-82-8 | 3.522±0.066^c^ | 5.636±0.127^a^ | 4.606±0.078^b^ | | |  |
| Allyl benzene | 300-57-2 | 3.034±0.166^a^ | 3.212±0.221^a^ | 2.984±0.235^a^ | | |  |
| Propylbenzene | 103-65-1 | 7.753±0.342^b^ | 9.341±0.049^a^ | 7.243±0.088^c^ | | |  |
| 1,3,5-Trimethylbenzen | 108-67-8 | 8.005±0.229^b^ | 9.788±0.078^a^ | 6.906±0.089^c^ | | |  |
| 3-ethyltoluene | 620-14-4 | 9.671±0.342^c^ | 16.396±0.076^a^ | 10.507±0.264^b^ | | |  |
| 1, 3-dimethyl-2-ethylbenzen | 002870-04-4 | 2.248±0.077^b^ | 4.125±0.089^a^ | — | | |  |
| 4-Ethyl-o-xylene | 934-80-5 | — | — | 7.603±0.165 | | |  |
| 1, 2-dimethyl-3-ethylbenzen | 933-98-2 | — | 12.247±0.462 | — | | |  |
| 1,2,4,5-Tetramethylbenzen | 95-93-2 | 5.812±0.075 | — | — | | |  |
| O-cymene (O-isopropylbenzene) | 527-84-4 | 7.303±0.141 | — | — | | |  |
| Meta-cymene (m-cymene) | 535-77-3 | 5.668±0.097 | — | — | | |  |
| Cymene (4-isopropyltoluene) | 99-87-6 | 20.639±0.277^c^ | 34.226±0.366^a^ | 22.515±0.285^b^ | | |  |
| 1- (1-METHYLETHENYL) -2-BENZENE | 5557-93-7 | 5.150±0.016^a^ | — | 3.626±0.042^b^ | | |  |
| 1,2,3,5-Tetramethylbenzen | 527-53-7 | — | 11.562±0.566^a^ | 5.816±0.074^b^ | | |  |
| Hydrocarbon |  |  |  |  | | |  |
| Ethylene oxide | 75-21-8 | — | 23.893±0.465 | — | | |  |
| Hexane | 110-54-3 | — | 4.848±0.094^b^ | 7.919±0.076^a^ | | |  |
| 4-Methylnonane | 17301-94-9 | 3.301±0.045^c^ | 5.332±0.062^a^ | 4.198±0.070^b^ | | |  |
| 2, 6, 6-Trimethyldecan | 62108-24-1 | — | — | 8.237±0.092 | | |  |
| 1-iodododecane | 4292-19-7 | 4.259±0.091^b^ | 8.584±0.131^a^ | — | | |  |
| 3-Methylnonane | 005911-04-6 | 12.667±0.133^c^ | 19.156±0.342^a^ | 17.724±0.452^b^ | | |  |
| 2, 2-Dimethylpentan | 590-35-2 | — | 3.314±0.068 | — | | |  |
| 2, 2, 7, 7-Tetramethyloctane | 1071-31-4 | 1.813±0.008^b^ | 4.509±0.164^a^ | — | | |  |
| Eicosane | 112-95-8 | 2.395±0.061^c^ | 4.464±0.121^a^ | 3.543±0.089^b^ | | |  |
| 3, 6-Dimethyloctane | 15869-94-0 | — | 18.302±0.445 | — | | |  |
| 1, 3-Dimethylcyclopentan | 2453-00-1 | 10.169±0.201 | — | — | | |  |
| 4-methyldodecane | 6117-97-1 | — | — | 5.157±0.106 | | |  |
| Hexadecane | 544-76-3 | — | 10.442±0.334 | — | | |  |
| 3-Ethyl-3-methylheptane | 17302-01-1 | — | 4.742±0.069 | — | | |  |
| Chloroform | 67-66-3 | — | 4.282±0.045^a^ | 4.326±0.064^a^ | | |  |
| Pentacosane | 629-99-2 | — | 4.041±0.066 | — | | |  |
| Undecane | 1120-21-4 | 13.979±0.777^c^ | 23.418±0.879^a^ | 19.176±1.201^b^ | | |  |
| N-undecane | 1120-21-4 | — | — | 3.276±0.098 | | |  |
| Heptacosane | 593-49-7 | — | — | 6.481±0.131 | | |  |
| N-undecane | 1120-21-4 | 6.774±0.242 | — | — | | |  |
| Tetracosane | 646-31-1 | — | 4.496±0.058 | — | | |  |
| 4-methyl octane | 2216-34-4 | — | 3.558±0.135^a^ | 2.195±0.059^b^ | | |  |
| 2, 6, 10-Trimethylpentadecan | 3892-00-0 | — | 3.723±0.067 | — | | |  |
| 2-methylundecane | 7045-71-8 | — | — | 5.724±0.166 | | |  |
| 3-methyldecane | 13151-34-3 | — | 12.726±1.453^a^ | 5.218±0.078^b^ | | |  |
| 3, 3-Dimethylhexan | 563-16-6 | — | — | 3.867±0.087 | | |  |
| N-dodecane | 112-40-3 | 39.361±2.664^c^ | 73.723±3.441^a^ | 41.681±2.686^b^ | | |  |
| Hentriacontane | 630-04-6 | — | 4.694±0.464 | — | | |  |
| Cyclododecane | 294-62-2 | — | 3.364±0.331 | — | | |  |
| 10-methylnonadecane | 56862-62-5 | — | 3.888±0.089 | — | | |  |
| 5-methyldecane | 13151-35-4 | 6.638±0.032^b^ | — | 10.496±0.873^a^ | | |  |
| 4-Methyldecane | 2847-72-5 | — | — | 5.947±0.483 | | |  |
| 4,5-Dimethylundecane | 17312-79-7 | — | 9.952±0.087 | — | | |  |
| 9-octylicosane | 13475-77-9 | — | 3.107±0.077 | — | | |  |
| 3,8-dimethyldecane | 17312-55-9 | 3.482±0.142^b^ | 5.339±0.183^a^ | — | | |  |
| 3,6-Dimethyldecane | 17312-53-7 | — | 6.988±0.355 | — | | |  |
| 10-methylicosane | 54833-23-7 | — | — | 6.740±0.241 | | |  |
| 4, 6-Dimethyldodecan | 61141-72-8 | 3.771±0.244 | — | — | | |  |
| 11-butyldocosane | 13475-76-8 | — | — | 4.164±0.243 | | |  |
| 5-Methyl-Undecane | 1632-70-8 | 2.237±0.068^b^ | 4.270±0.112^a^ | — | | |  |
| Diethyl (decyloxy) -Borane |  | 7.322±0.198^c^ | 13.305±0.246^a^ | 8.181±0.227^b^ | | |  |
| 9-octyl heptadecane | 7225-64-1 | — | 8.783±0.445 | — | | |  |
| 4,8-Dimethylundecane | 17301-33-6 | — | 4.139±0.132 | — | | |  |
| 5,14-dibutyldecane | 55282-13-8 | — | — | 3.462±0.365 | | |  |
| 2-methyltetracosane | 1560-78-7 | 3.546±0.049 | — | — | | |  |
| 3-Methyl-Eicosane | 6418-46-8 | — | 6.859±0.078 | — | | |  |
| TETRACONTANE, 3,5,24-TRIMETHYL | 55162-61-3 | — | 3.215±0.204 | — | | |  |
| Nonylcyclopentane | 2882-98-6 | 3.650±0.343^b^ | 7.282±0.088^a^ | 2.360±0.076^c^ | | |  |
| 5-Methyltricosane | 22331-09-5 | 3.741±0.226 | — | — | | |  |
| N-tridecane | 629-50-5 | 33.282±1.887^b^ | 60.578±3.645^a^ | 31.784±1.091^c^ | | |  |
| N-decane | 124-18-5 | — | 4.334±0.254 | — | | |  |
| Octadecane | 593-45-3 | — | 3.589±0.099 | — | | |  |
| Heptyl cyclohexane | 5617-41-4 | 1.920±0.074^b^ | 4.129±0.105^a^ | 1.864±0.112^b^ | | |  |
| 2-Bromododecane | 13187-99-0 | 6.093±0.209^a^ | — | 3.080±0.065^b^ | | |  |
| Hexatriacontane | 630-06-8 | — | 7.671±0.455 | — | | |  |
| N-tetradecane | 629-59-4 | 7.716±0.226^c^ | 11.560±0.231^a^ | 10.301±0.331^b^ | | |  |
| 2, 6, 10, 14-Tetramethylheptadecan | 18344-37-1 | — | 3.821±0.226 | — | | |  |
| Benzocyclobutene | 694-87-1 | 253.391±3.445^c^ | 306.666±3.245^b^ | 313.973±3.665^a^ | | |  |
| Styrene | 100-42-5 | 19.803±0.287^b^ | 23.650±0.365^a^ | 17.627±0.447^c^ | | |  |
| Cyclooctatetraene | 629-20-9 | 5.074±0.034^b^ | 6.063±0.042^a^ | | 3.967±0.093^c^ | |  |
| Pinene | 7785-70-8 | 8.223±0.022^b^ | 13.697±0.132^a^ | | 7.918±0.142^c^ | |  |
| 3-ene | 13466-78-9 | 12.825±0.556^c^ | 28.237±0.787^a^ | | 16.576±0.464^b^ | |  |
| Alpha-terpinene | 99-86-5 | 2.811±0.088^c^ | 4.742±0.077^b^ | | 5.622±0.064^a^ | |  |
| β-phellandrene | 555-10-2 | 7.875±0.257 | — | | — | |  |
| 4 (5) -ene | 29050-33-7 | — | — | | 26.326±0.532 | |  |
| Ocimene | 13877-91-3 | — | — | | 4.814±0.095 | |  |
| γ-terpinen | 99-85-4 | 10.683±0.168^c^ | 18.336±0.165^a^ | | 11.052±0.163^b^ | |  |
| Terpinolene | 586-62-9 | 11.691±0.202^b^ | 17.643±0.404^a^ | | 9.954±0.434^c^ | |  |
| 4, 5-Dimethyl-1-hexen | 16106-59-5 | — | 4.502±0.080 | | — | |  |
| 2, 4, 4-Trimethyl-1-hexen | 51174-12-0 | 5.150±0.076^b^ | 11.237±0.152^a^ | | 4.657±0.043^c^ | |  |
| 1-Tridecene | 2437-56-1 | 2.257±0.115^b^ | 4.850±0.221^a^ | | — | |  |
| (+) -Longifolene | 1137-12-8 | 4.765±0.121^b^ | 7.928±0.084^a^ | | 4.261±0.063^c^ | |  |
| Longifolene | 475-20-7 | 5.481±0.096^b^ | 8.679±0.087^a^ | | 4.941±0.049^c^ | |  |
| (+) -Limonene | 5989-27-5 | 20.639±0.543^c^ | 40.954±0.645^a^ | | 33.024±0.022^b^ | |  |
| Alpha-thujene (a-thujene) | 002867-05-2 | 6.712±0.103^b^ | 8.746±0.085^a^ | | — | |  |
| Cadina diene | 29837-12-5 | — | — | | 12.419±0.187 | |  |
| alpha-bulnesene | 3691-11-0 | — | 6.975±0.083^a^ | | 3.452±0.029^b^ | |  |
| Beta-himachalene | 1461-03-6 | — | 3.700±0.044 | | — | |  |
| 1-butoxy-3-methyl-2-butene | 22094-02-6 | 3.392±0.017 | — | | — | |  |
| Other |  |  |  | |  | |  |
| 1-hydroxymethylimidazole | 51505-76-1 | — | — | | 3.094±0.052 | |  |
| 2-pentylfuran | 3777-69-3 | 26.905±0.686 | — | | — | |  |
| Indane | 496-11-7 | — | 5.577±0.076^a^ | | 3.194±0.043^b^ | |  |
| 2, 4, 5-Trimethylthiazol | 13623-11-5 | — | 7.079±0.064^a^ | | 2.862±0.022^b^ | |  |
| Clemastine | 15686-51-8 | — | 6.817±0.033 | | — | |  |
| Cis-2,6-dimethylpiperazin | 21655-48-1 | — | 4.197±0.054 | | — | |  |
| 1, 4-Dimethyl-1, 2, 3, 4-tetrahydronaphthalen | 4175-54-6 | — | 6.069±0.085 | | — | |  |
| 1, 5-Dimethyl-1, 2, 3, 4-tetrahydronaphthalen | 21564-91-0 | — | 6.389±0.076 | | — | |  |
| 5-Vinyl-2-methylpyridin | 140-76-1 | — | 7.644±0.073 | | — | |  |
| 5H-Naphtho[2,3-b]carbazole | 248-96-4 | 3.945±0.057 | — | | — | |  |
| 4-propyl-2,3-dihydro-1H-indene | 92013-16-6 | 4.264±0.022^a^ | — | | | 3.606±0.048^b^ | |
| 1,2,3,3a,4,7,8,8a-Octahydro-1,4,6-trimethyl-4,7-ethanoazulene | 65128-08-7 | 4.330±0.061 | — | | | — | |
| 2-methyl-Adenosine | 16526-56-0 | 14.174±0.112 | — | | | — | |
| 2-Chloro-4-(4-methoxyphenyl)-6-(4-nitrophenyl)pyrimidine | 63673-76-7 | — | 19.852±0.221^a^ | | | 10.106±0.101^b^ | |
| 6-Ethyltetralin | 22531-20-0 | — | 3.045±0.018 | | | — | |
| 2-Benzo[1,3]dioxol-5-yl-8-methoxy-3-nitro-2H-chromene |  | — | 3.632±0.042 | | | — | |

_Note: Contents less than 3 μg/100g are not shown;-indicates that the substance is not detected._ ^a,b,c^ _Different letters in the same row indicate significant differences at p ≤ 0.05. Values are the mean± standard deviation of three independent replicates (n = 3)._
